# Supplementary material for: The language of marketing hyperbole and consumer perception–The case of Glasgow
Source: PLoS One. 2023 Dec 20;18(12):e0295132. doi: 10.1371/journal.pone.0295132 (PMC10732446; doi:10.1371/journal.pone.0295132)
Supplement: S1 Appendix — (DOCX) [file pone.0295132.s001.docx]

# Appendix 1

**In Other Words: A short word association game**

The next questions will present you with the name of a city with the request to write as many keywords as you can think of that you associate with it.

The survey will progress to the next question when the timer expires in 25 secs. Please use a new line (hit Return) for each association, for example, if presented with the word BIRD you might write:

flying

feathers

the word

yellow

ducks

When you hit NEXT the timer will begin.

Q1: [TIMED ASSOCIATION] In the next 25 seconds, describe what you associate with the **CITY of GLASGOW**.

Q2: [TIMED ASSOCIATION] In the next 25 seconds, describe what you associate with the **PEOPLE of GLASGOW**.

Q3: Have you ever been to Glasgow? (Yes|No)

If NO SKIP STRAIGHT to Q4.

IF YES:

A: Which best describes your relationship to GLASGOW? Check all that apply. *

- Resident
- Former resident
- Work here
- Have visited (work)
- Have visited (study)
- Have visited (tourist)
- In transit only (i.e. passed through the city, or stayed only very briefly).
- Other - please specify

B: Which aspects of Glasgow City appeal most to you? *

- Museums and galleries
- Nightlife
- Music
- Food and hospitality offers
- Retail
- Built heritage (old buildings)
- Conference and meetings facilities
- Sport
- Other - Please state

Q4: Which of the following best describes the highest level of education obtained? *

- Less than secondary school
- Secondary school
- College/further education
- University/higher education
- Advanced degree (Master's, Ph.D., M.D.)
- Other - Please specify

Q5: Which of the following best describes your current employment? *

- Full time
- Part time
- Not employed
- Student
- Retired
- Disabled
- Other - Please specify

Thank you.
