# Supplementary material for: The language of marketing hyperbole and consumer perception–The case of Glasgow
Source: PLoS One. 2023 Dec 20;18(12):e0295132. doi: 10.1371/journal.pone.0295132 (PMC10732446; doi:10.1371/journal.pone.0295132)
Supplement: Attachment — Submitted filename: Editor COMMENTS to original submission PONE-D-23-11026 20230707.pdf [file pone.0295132.s002.pdf]

Overall:

Please improve the clarity throughout your entire text. Currently, there are many unclear sentences like “Linguistic associations may include common properties of a city such as town or busy (39), while highly idiosyncratic properties might be generated that are very different and capture personal experiences rather than generic descriptors (40).” – different from what? what is the meaning of “while” in this sentence? Are those “idiosyncratic properties” something distinct to linguistic associations?

Abstract:

The Abstract is unclear, the language should be improved. For example,

“had greater variance and uniqueness” – please clarify what the variance of the word and its uniqueness is

“The order of words may represent a greater or lesser focus on systems dealing with linguistic associations versus situated simulations.” - unclear

“Aaker-Dimensions” – should be explained for readers unfamiliar with brand personality theory

“to study perception” – whose perception? Of what?

Monotonous style:

“Additionally, a logistic regression analysis was conducted between the binary variable never visited Glasgow and the Aaker-Dimensions. The results showed that the Aaker-Dimensions had a significant relationship with the binary variable never visited Glasgow.”

“practical, fast, and replicable methodology” – it should be rather “method.”

Introduction:

Please outline the aim of your research by explaining the gap you want to bridge and formulating you research question.

Hypotheses:

“A key premise of this paper is the idea that “you shall know a word by the company it keeps”” – please clarify.

What is the justification for H2?

Exploratory analyses:

What is the motivation of those analyses? It is not clear to me after reading the introductory part of your manuscript.

Conclusion:

What is the theoretical and practical importance of the results of your hypothesis testing?
