# Supplementary material for: The language of marketing hyperbole and consumer perception–The case of Glasgow
Source: PLoS One. 2023 Dec 20;18(12):e0295132. doi: 10.1371/journal.pone.0295132 (PMC10732446; doi:10.1371/journal.pone.0295132)
Supplement: Attachment — Submitted filename: PONE-D-23-11026R2 Glasgow COMMENTS.pdf [file pone.0295132.s005.pdf]

Thank you for your response to the concern of Reviewer 1. However, the communication in your manuscript is still far from being acceptable. There are many imprecise or unclear expressions. I believe most of them are caused by language mistakes (e.g., in the sentence "The extent to which word associations varied by participants' relationship to Glasgow was identified, in terms of Aaker's brand personality scale, an extension of personality research to brands and destinations.", why did you put the comma before "interns"?). Another example: you misspelled the word "collocation" (row 61). You write "Covid" instead of "COVID-19". Your manuscript should be rigorously proofread to be accepted. Those problems create the impression that your manuscript is not digested and also lead to serious problems with understanding your key statements. For example, in H1, you state that "Word associations written later by the respondent in the timed response are more likely to be situated simulations (SS) than those earlier language associations (LA)…". Why did you put "those earlier" in that statement? If I understand your idea correctly, it should be, "Word associations written later by the respondent in the timed response are more likely to be situated simulations (SS) than language associations (LA)." Next, you write, "SS is operationalised in terms of semantic distance, with SS predicted to be more distant from one another (variance across terms generated) and the core concept of city (extended to tests of Scotland and Glasgow as sub-categories) than LA". This sentence is completely unclear. What does it mean "from one another"? Again, if I understand your idea correctly, it should be something like, "Word associations written later are predicted to be more distant from each another." Maybe discussing that operationalization in the method section would be clearer.

It is unclear what the expression "Aaker dimensions are sufficient" means (row 321). It is also unclear what variables were involved in your Exploratory Factor Analysis. It is not explained in the Method section. It remains unclear in the Results section. E.g., how is the variable corresponding to the item "park" defined at the respondent level? (I suppose that respondents are units of your analysis).

You state that your exploratory analyses aim to address the research questions, "What other terms emerge in descriptions of Glasgow and its people, and how might these be used to extend of enhance the Aaker model?". However, in row 321, you declare another aim of your exploratory analyses ("To see if the Aaker personality dimensions were sufficient.). This is inconsistent. By the way, the expression "other terms" (row 343) is unclear (other vs. what?).

In the Results section, when you report on differences, please support them with statistical tests (e.g., rows 346-350). Instead of "$p = 0.000$," it should be "$p < 0.001$."

The sentence "It is the absence of this powerful association in all of the destination marketing examined that is perhaps most telling and reaffirms the distance between marketing hyperbole and perception" is completely unclear. E.g., what do you mean by "powerful association" and destination marketing examined"?

Your Discussion section is dominated by exploratory findings, which seem disconnected from the main aim of your study (see, e.g., your Abstract). In general, my impression is that your study aims to investigate two issues related to destination image: (1) LA vs. SS associations in time and (2) the differentiation of destination personality between destination inhabitants and non-inhabitants. If so, two things should be improved. First, the above aims should be reflected in the title, which can refer to Glasgow as an example (case) you use. Second, the readers would like to see the research gap: how your findings advance the literature. Were those two issues studied before in the destination context? You do not explain that. For example, in the theoretical background section, you only say, "The present study employs LASS as a theoretical lens to both categorise and interpret the properties generated and provide an additional opportunity to test predictions made by the theory." In the

Conclusions section, you say that “the paper proposed analysing word order in terms of semantic distance, as a means of identifying the activation of different systems in word association tasks.” Does it mean that some previous studies on tourist destinations already evidenced the LA vs. SS associations in time, and your contribution is only the new method?
