# Supplementary material for: The language of marketing hyperbole and consumer perception–The case of Glasgow
Source: PLoS One. 2023 Dec 20;18(12):e0295132. doi: 10.1371/journal.pone.0295132 (PMC10732446; doi:10.1371/journal.pone.0295132)
Supplement: Attachment — Submitted filename: Response to Reviewers.docx [file pone.0295132.s003.docx]

|  | **PLOS One** | **Research Team** |
| --- | --- | --- |
| Editorial comments | | |
| Overall | Please improve the clarity throughout your entire text. Currently, there are many unclear sentences like “Linguistic associations may include common properties of a city such as town or busy (39), while highly idiosyncratic properties might be generated that are very different and capture personal experiences rather than generic descriptors (40).” – different from what? what is the meaning of “while” in this sentence? Are those “idiosyncratic properties” something distinct to linguistic associations? | Section 2.3, changed example cited here “Linguistic associations may include common properties of a city…“ to  “Linguistic associations may include properties conceptually common to a city such as *large* or *busy* (39), as well as properties that capture unique personal experiences, for example *the pie-shop under the bridge* (40).  Also made the following paragraph clearer removing one superfluous citation and referencing the “*pie shop*” in relation to the situated simulation concept.  Added a new chapter in methods to describe the procedure for the exploratory factor analysis:  “3.7 Exploratory factor analysis  To see if the Aaker personality dimensions were sufficient, an exploratory factor analysis (...) fewer than ten occurrences were removed."  Removed first sentence in 4.3.2. as it is already explained in Methods: “Logistic regression analysis between (…) distance measures.” Also added more clarity when stating the regression results “A logistic regression revealed that participants (…) higher for those describing Glasgow as sophisticated.”  More accurate language in 4.3.1, as the semantic distance relates to the words themselves, not the core concepts: “last words were on average more semantically distant from the words Scotland (t=5.43, p=0.000, d=0.22), city (t=5.88, p=0.000, d=0.22) and town (t=8.07, p=0.000, d=0.31) than first words”  Improved clarity in 3.5: “The difference in the average distances between unique first words and the average distances between last words were then tested using an independent sample t-test.”  Throughout the text, Aaker-dimensions had also been regarded as “categories”. This was now standardized, speaking only of “Aaker-dimensions” or, in its short form, “dimensions”. |
| Abstract | The Abstract is unclear, the language should be improved. For example, “had greater variance and uniqueness” – please clarify what the variance of the word and its uniqueness is “The order of words may represent a greater or lesser focus on systems dealing with linguistic associations versus situated simulations.” - unclear “Aaker-Dimensions” – should be explained for readers unfamiliar with brand personality theory “to study perception” – whose perception? Of what? | Changed to:  “Evidence was found in support of dual-processing theory suggesting associations based on lived experiences may be generated later in a word association tasks, while common linguistic associations (e.g. synonyms, antonyms, hierarchies etc.) tend to be generated earlier. Additionally, a logistic regression analysis revealed a significant relationship between several of the Aaker-dimensions of brand personality, and the relationship to Glasgow.” |
|  | “Additionally, a logistic regression analysis was conducted between the binary variable never visited Glasgow and the Aaker-Dimensions. The results showed that the Aaker-Dimensions had a significant relationship with the binary variable never visited Glasgow.”  “practical, fast, and replicable methodology” – it should be rather “method.” | Simplified sentence in the Abstract:  “Additionally, analyses revealed a significant relationship between several of the Aaker-dimensions of brand personality, and the relationship to Glasgow”  Changed. |
| Monotonous style | Look for monotonous or lengthy writing style. | 3.6.: if the semantic distance was below a certain threshold and hence (…) assigned a “1” for the Aaker-Dimensions of Competence and Ruggedness, and a “0” for Sincerity, Excitement and Sophistication.  Improvements to monotonous style and simplification in several parts of the result section:  4.2.1 “Positive descriptors such as friendly, funny, and fun, as well as Adjectives referring to the more rough features of Glasgow such as cold, rough and busy, are among the most common associations. Accents, football and culture are nouns frequently mentioned in the context of Glasgow”  4.2.2: “above the age of 55, for whom culture and football are much more common associations than for younger ages.”  4.2.3: “*football* is by far the most frequently mentioned term for men , and *cold* the second most frequent, while the top two are reversed for women and much closer in frequency.”  4.2.4: “It was hypothesized that (residents (self) would use a different linguistic style to people who have never visited (other)”  3.3. Combined two sentences to reduce repetition. Improved clarity: “Furthermore, all plural words were converted to their singular form. As there was no standardized format to identify bigrams or trigrams, such as hard-working or down-to-earth, all possible combinations of bigrams and trigrams were collected programmatically.  Many smaller changes made throughout the text, see tracked changes. |
| Intro | Please outline the aim of your research by explaining the gap you want to bridge and formulating you research question. | Introduction enhanced:   “Defining and building a brand requires a clear understanding of perception in terms of the destination and its people who live there, across various demographics and relationships to the destination, yet such comparisons are rare, and tourists remain the subject of most tourism research, with local perceptions poorly represented [9]. Digital trace data generated when interacting with online media, such as Twitter, Facebook, Trip Advisor, Booking.com and similar are a rich source of such perception, however, they are also archival and observational [10–12], and not the results of a controlled process. Given a working theory, however, can help guide the generation of insights at scale in social media, and one promising means of achieving such a foundation is through word associations, the space those words inhabit, and the company they keep (i.e. co-occurrence and colocation) [13,14].  Perceptions of Glasgow, and the ways in which those perceptions vary by way of the strength of the relationship to the city and its people, had to date, never been explored at scale, and represent a critical opportunity for destination marketers, especially given the novel challenges emerging from a post-Covid world.” |
| Hypotheses | “A key premise of this paper is the idea that “you shall know a word by the company it keeps”” – please clarify.  What is the justification for H2? | Section 2.6.  Modified: A key premise of this paper is the idea that “you shall know a word by the company it keeps” (53). The company a word keeps is defined by its semantic distance and location relative to other words as derived from large text corpuses, also known as word embeddings. For example, the word *bee* will often be found close to words such as *hive*, and *sting*, and less frequently close to the word *metallurgy*. Word embeddings allow for the meaning of words to be represented mathematically, and to facilitate computational approaches.  To address this, Section *2.5 Self versus Other: Residents and Tourists* has been added, and hypotheses shifted to 2.6, to focus specifically on the role of self and other in destination perception and provide greater context for H2.  Also preceded “H2: Self-perception (residents) of Glasgow city and people…” in 2.6 with:  “As discussed in Section 2.5, there is considerable evidence to suggest that how residents and visitor perceive a destination may vary substantially. Where the Aaker scale seeks to provide a universal instrument to capture the essence of a destination, the present study hypothesizes that a destination may have two or even several personalities, when segmenting by relationship to the destination. It follows, therefore, that:…” followed by the hypothesis. |
| Exploratory analyses | What is the motivation of those analyses? It is not clear to me after reading the introductory part of your manuscript. | Following guidance around confirmatory studies and the ability to include additional findings of interest, the exploratory analyses were an expected result of looking for trends that may not have been included in the hypotheses, and yet which might still be valuable for future research. This follows recommendations from PLOS One and other open science sites, whereby “Choosing to preregister a confirmatory study doesn’t prevent you from conducting related exploratory research or post-hoc analysis. You reap the benefits of increased trust and reproducibility, with no risks or limitations to your future research.” (<https://plos.org/open-science/preregistration/>). We have added a note in the study to help make this choice clearer.  Section 2.6 extended with:  “Following guidance around the preregistration of confirmatory studies regarding the potential value of including subsequent exploratory research or post-hoc analysis (e.g. https://plos.org/open-science/preregistration) in addition to these pre-registered hypotheses, relationships and categories beyond the Aaker dimensions were explored, including differences across demographics as both original insights and opportunities for future research.” |
| Conclusion | What is the theoretical and practical importance of the results of your hypothesis testing? | Made substantial changes to Section *7 Conclusion*, providing a closer tie to theory in terms of LASS and Aaker’s brand personality scale for destination marketing, and elaborating the practical opportunities of using word associations and NLP at scale. Also made reference to how the knowledge of a spectrum of perception by relationship may inform destination marketers and provides simple tools to gather and process perception data in terms of those variations in relationship.  Details provided in Reviewer Suggestions 5 and 6 as the same comments were made by the reviewer. |
| 1 | Please ensure that your manuscript meets PLOS ONE's style requirements, including those for file naming. The PLOS ONE style templates can be found at  https://journals.plos.org/plosone/s/file?id=wjVg/PLOSOne_formatting_sample_main_body.pdf and  https://journals.plos.org/plosone/s/file?id=ba62/PLOSOne_formatting_sample_title_authors_affiliations.pdf | Done. |
| 2 | Please provide additional details regarding participant consent. In the ethics statement in the Methods and online submission information, please ensure that you have specified (1) whether consent was informed and (2) what type you obtained (for instance, written or verbal, and if verbal, how it was documented and witnessed). If your study included minors, state whether you obtained consent from parents or guardians. If the need for consent was waived by the ethics committee, please include this information. | Within the Methods section, a new Section 3.1 Ethics has been added with the following information:   “An ethics application was made and approved by the **Glasgow School *for* Business and Society** (GSBS) ethics committee to ensure all due ethical considerations were taken in the study design, as the research study involved human participants. Participants had to be 18 years or older, and so no minors were involved in the study. The following written explanation of the project was provided through the Prolific service advertisement at the start of the survey, explaining the aim and benefits of the study, as well as the expected remuneration:  *The aim of the study is to explore the perception of Scottish cities through word associations in order to help local governments better understand urban locations. If you participate, you will be asked to write as many words as you can think of for two aspects of one Scottish city, in 25 seconds. The association task is then followed by a short series of questions to understand your relationship to the city, and some basic demographics. All in all, the study should take no longer than a few minutes to complete (7-9 questions in total).*  Users were asked to provide their Prolific ID in order to obtain demographic information they had previously provided to the service. Any personal information linked to this ID was not stored with the survey data and was completely inaccessible to the research team) and no additional personally identifiable information was requested or obtained by the research team, rendering the survey data anonymous. A box to check for participants to provide consent prior to participation was also provided.” |
| 3 | Please include your full ethics statement in the ‘Methods’ section of your manuscript file. In your statement, please include the full name of the IRB or ethics committee who approved or waived your study, as well as whether or not you obtained informed written or verbal consent. If consent was waived for your study, please include this information in your statement as well. | Section *3.1 Ethics* now added. |
| 4 | We note that you have stated that you will provide repository information for your data at acceptance. Should your manuscript be accepted for publication, we will hold it until you provide the relevant accession numbers or DOIs necessary to access your data. If you wish to make changes to your Data Availability statement, please describe these changes in your cover letter and we will update your Data Availability statement to reflect the information you provide. | Data available here: <https://osf.io/amscb/>. This has also been referenced in the paper at the Data Availability Statement and in Section 3 “The following section provides an overview of our methods. In support of both validation and replication by others, the full data and Python notebooks are available here: <https://osf.io/amscb/>.” |
| Reviewer 1 | | |
| 1 | The full details of word association procedures should be presented. | Section 3.6 contains additional detail now, while all methods and tests are available in the supplementary materials (specifically the Python notebook). |
| 2 | The authors must be clear of the sample characteristic. For example, among 1219 participants, how many are residing residents, first-time tourists and repeated tourists. Based on the current report, my understanding is 277 residing residents, 418 visited tourists, and 524 never been to Glasgow. Please do correct me if I am wrong. | That is correct, though there was a mistake in the original body of the text, should be 276, and 419 not 277 and 418. Now corrected in 3.1.  Section 4.1 Table 1 has the detailed descriptive stats.   - Never visited: 524 (43%) - Visited but never lived there: 419 (34.4%) - Lived or living there: 276 (22.6%) |
| 3.1 from 3.3.2 | Survey is referring to the instrument that should be placed before 3.1 participants. | The survey in Appendix 1 is referenced before 3.1 Participants with the following text:  “The survey instrument used in the study can be found in Appendix 1.” |
| 3.3 | Natural Language Processing is based who’s suggestion? | Section 3.3 enhanced with:  “Natural Language Processing (NLP) is a common approach to the automated analysis of large bodies of text, that has been frequently applied to the categorisation of words in word association tasks (for example 54–58) and is a frequently used method for exploring human cognition and perception, such as creativity (59) stereotypes (60) and social biases (61). Most immediately relevant to the present study, it has also been used to explore tourist perception in blogs (62) and general tourism data mining (63).” |
| 5. | The authors must address how does this study value add to the existing tourism literature. | The was addressed previous in response to the editor, who made similar suggestions. “What is the theoretical and practical importance of the results of your hypothesis testing?”  Specifically, two paragraphs were added to the conclusion section for each of the contributions to the grounded cognition-based theory of LASS, and the Aaker brand personality dimensions.  Section *7: Conclusion*  LASS: “The study contributes to theory and practice in several meaningful ways. In terms of theory, the paper proposed analysing word order in terms of semantic distance, as a means of identifying the activation of different systems in word association tasks (H1). Evidence was found in support of the idea that both greater average semantic distance from core concepts and greater uniqueness in terms generated may provide a workable proxy for identifying active systems over costly approaches such as fMRI scans, representing a contribution to LASS theory, and grounded cognition.”  Aaker: “With regards to Aaker’s theory of brand personality, the study contributes to nascent research into the multiple personalities a destination may possess, when considered from the perspective of an observer’s relationship to the city and offers an opportunity to extend existing theory through recognition that relationships to a destination may substantially impact perception (H2). The study found evidence for a tale of two cities, and while the differences found were not as revolutionary as those in Charles Dickens’ novel, residents appear to inhabit a subtly different Glasgow to that experienced by visitors or perceived by non-visitors. The extent to which this may be true for other destinations, and particularly urban destinations, offers a rich opportunity to extend existing theory.” |
| 6. | Also, how could these findings offer insights for practitioner in managing Glasgow destination image. This must be addressed to reflect the usefulness of the results on to the real-life situation. | The was also raised by the editor. Further detail provided:   “Indeed, variations in perception from non-visitors to clearly illustrates a need to target these groups according to their familiarity with the city, and the presented methods provide marketers with tools to quickly obtain a snapshot into targeted perceptions.  Despite the short time investment required to gather data for this study, the richness of insights and interpretation that could be derived were, arguably, disproportionately high with evidence for the perception drawn from both immediate associations and those derived from lived experience of the city  The methods presented in this study offer destination marketers and researchers a simple, replicable approach to obtaining qualitative perception data in a controlled manner, at scale. Furthermore, the use of NLP detailed provides a fast and cost-effective way of matching associations to either predefined categories such as the Aaker dimensions, or any other terms of interest. In the case of Glasgow, these methods could be used to measure the effectiveness of campaigns such as Scotland with Style, by measuring the semantic distance of terms generated by respondents to the word style, or they could be used to identify potential terms for future marketing campaigns.” |
