# Supplementary material for: The language of marketing hyperbole and consumer perception–The case of Glasgow
Source: PLoS One. 2023 Dec 20;18(12):e0295132. doi: 10.1371/journal.pone.0295132 (PMC10732446; doi:10.1371/journal.pone.0295132)
Supplement: Attachment — Submitted filename: Response to Reviewers (20230827).docx [file pone.0295132.s004.docx]

|  | **PLOS One** | **Research Team** |
| --- | --- | --- |
| *Editorial comments* | | |
| General | The clean version of the manuscript differs from the mark-up version (e.g., look at the Abstract; the last sentence in the mark-up version is absent in the clean version).  Therefore, I cannot evaluate the communication of your revised manuscript. Please fix it. | *Our apologies, it seems we attached the older version (from April), should have been from July 30 (Revised Manuscript with Track Change.docx).*  *Now attached the prior mark-up version from July 30 as supporting information, and a new mark-up version form the latest version of the manuscript.* |
| Analysis | 2. Exploratory analysis: It looks like this analysis aims to answer some additional research questions (you mentioned that in row 659 of the mark-up version). Please explicitly state those questions before presenting that analysis.  Were your hypotheses preregistered? If so, please specify the preregistration platform. If not, please do not call them so (e.g., row 659 of the mark-up version). | *There were no further research questions, only observations and speculation regarding some other trends discovered during analysis. For clarity, we have now added detail to the “new questions” notes on in 434-435.*  *Specifically, what other terms emerge in descriptions of Glasgow and its people, and how might these be used to extend of enhance the Aaker model? Results to these questions provided in 4.5.1 and 4.5.2.*  Pre-registration and rationale for exploratory analyses (response to previous reviewer comments) was noted in lines 208-211:  “*Following guidance around the preregistration of confirmatory studies regarding the potential value of including subsequent exploratory research or post-hoc analysis (e.g. https://plos.org/open-science/preregistration) in addition to these pre-registered hypotheses, relationships and categories beyond the Aaker dimensions were explored, including differences across demographics as both original insights and opportunities for future research*.”  That said, the link to the pre-registration was missing. Now added.  **NEW**: Line 210: “…in addition to these pre-registered hypotheses (<https://osf.io/5zxfp>)”. |

| *Reviewer Comments* | | |
| --- | --- | --- |
| Study Design | Thank you for the revision while I appreciate the authors' efforts, the results of implicit association require further explanation. The calculation and grouping of implicit association results is missing. For instance, Harvard Implicit does indicate the categories for each implicit association. Based on reaction time and further calculation, each respondent can be classified into one of the groups. Hence, what is corresponding calculation and grouping for this research. For example, -0.15 to 0.15 is noted as neutral; 0.15 -0.349 is slightly positive; 0.35 - 0.649 is moderate while o.65 to 2.0 is strongly. Vice versa for the negative values. So, what is distribution of such categorization？ | Thank you for your notes. The study does not employ an IAT. It is a timed (or rather time limited) free word association study. |
| Sampling | Next, for the sample, about half of them have never been to Glasgow, and their perceptions do exert impacts on the overall results. How does the author account for this interference during analysis? | That was a desired aspect of the study, to identify differences in responses across those who had been to Glasgow (considering former residents an residents, then visitors, then those who had never been to Glasgow. This is captured in H2 (lines 253-255 in the most recent document):  *“H2: Self-perception (residents) of Glasgow city and people, and other-perception (visitors and never-visited) will differ significantly across coded brand dimensions. Terms generated by respondents will vary by relationship to Glasgow in terms of their Aaker distribution.”*  This is discussed at each stage of the analysis and in the related notebooks. |
