# Supplementary material for: The language of marketing hyperbole and consumer perception–The case of Glasgow
Source: PLoS One. 2023 Dec 20;18(12):e0295132. doi: 10.1371/journal.pone.0295132 (PMC10732446; doi:10.1371/journal.pone.0295132)
Supplement: Attachment — Submitted filename: Response to Reviewers 20231018.docx [file pone.0295132.s006.docx]

|  |  | **DATE: October 18, 2023** |
| --- | --- | --- |
|  | **PLOS One** | **Research Team** |
| Editorial comments | | |
| Proofreading | There are many imprecise or unclear expressions. I believe most of them are caused by language mistakes (e.g., in the sentence “The extent to which word associations varied by participants’ relationship to Glasgow was identified, in terms of Aaker’s brand personality scale, an extension of personality research to brands and destinations.”, why did you put the comma before “interns”?). Another example: you misspelled the word “collocation” (row  61). You write “Covid” instead of “COVID-19”. Your manuscript should be rigorously proofread to be accepted. Those problems create the impression that your manuscript is not digested and also lead to serious problems with understanding your key statements. | The manuscript has been proofread and copyedited for clarity. We hope there are no further issues, however, please flag any should you find them and we will correct (see the track changes version of the manuscript for all changes made). |
| Clarity of Hypotheses | For example, in H1, you state that “Word associations written later by the respondent in the timed response are more likely to be situated simulations (SS) than those earlier language associations (LA)…”. Why did you put “those earlier” in that statement? If I understand your idea correctly, it should be,  “Word associations written later by the respondent in the timed response are more likely to be situated simulations (SS) than language associations (LA).”  Next, you write, “SS is operationalised in terms of semantic distance, with SS predicted to be more distant from one another (variance across terms generated) and the core concept of city (extended to tests of Scotland and Glasgow as sub-categories) than LA”. This sentence is completely unclear. What does it mean “from one another”? Again, if I understand  your idea correctly, it should be something like,  “Word associations written later are predicted to be more distant from each another.”  Maybe discussing that operationalization in the method section would be clearer. | This has been rewritten for clarity.  In 2.6 Hypotheses:  “H1: Word associations written later are predicted to be more distant from the city and each other.”  AND  “Word associations generated later in the word association task by the by the respondent are more likely to be situated simulations (SS) than language associations (LA).”  H1b also rewritten as “Word associations written later are predicted to be more distant from each another”  “Specifically, SS are predicted to be more distant from the core concept of city (extended to tests of Scotland and Glasgow as sub-categories) than LA, and to have higher variability (i.e., terms generated later will be more unique from one another in terms of average semantic distances than the average distances calculated for LA).”  Also added the new working to the method section (3.5).  “H1b stated that **“**Word associations written later are predicted to be more distant from each another**”.** To test **this hypothesis**, the variance of associations was calculated within the group of all unique first, and all unique last words, respectively.” |
| Exploratory analyses | It is unclear what the expression “Aaker dimensions are sufficient” means (row 321). It is also unclear what variables were involved in your Exploratory Factor Analysis. It is not explained in the Method section. It remains unclear in the Results section. E.g., how is the variable corresponding to the item “park” defined at the respondent level? (I suppose that respondents are units of your analysis). | Now changed in Section 3.7  “To see if the Aaker personality dimensions were utilised by the sample in their timed responses, an exploratory factor analysis with varimax rotation was run separately for both city and people as a method for investigating the co-occurrence of words. This was achieved by creating a binary column for each term of the word association task (for example, “park”), to indicate whether a participant had, or had not used the term. Then, to ensure that the words used had sufficient weight and relevance, words with fewer than ten occurrences were removed. Words that tend to appear together in the same response, were placed into a common category. |
|  | You state that your exploratory analyses aim to address the research questions, “What other terms emerge in descriptions of Glasgow and its people, and how might these be used to extend of enhance the Aaker model?”. However, in row 321, you declare another aim of your exploratory analyses (“To see if the Aaker personality dimensions were sufficient.). This is inconsistent. By the way, the expression “other terms” (row 343) is unclear (other vs. what?). | Removed this inconsistency. Now aligned with:  “In addition to the pre-registered hypotheses, additional ad hoc analyses were conducted to explore what other terms outside of the Aaker dimensions may have emerged in descriptions of Glasgow and its people, and how might these be used to extend the Aaker model.”  Also Section 7: Conclusions a sentence added: “In subsequent exploratory analyses, possible extensions to the Aaker dimensions were also identified for Glasgow.” |
| p-value formats | In the Results section, when you report on differences, please support them with statistical tests (e.g., rows 346-350). Instead of “p = 0.000,” it should be “p < 0.001.” | This has been corrected. |
| Discussion | The sentence “It is the absence of this powerful association in all of the destination marketing examined that is perhaps most telling and reaffirms the distance between marketing hyperbole and perception” is completely unclear. E.g., what do you mean by “powerful association” and destination marketing examined”? | This statement was indeed sweeping and unclear, it has been reworded (Section 5.3):  “The absence in the destination marketing literature of this common word association between football and Glasgow further raises questions about the distance between marketing hyperbole and the perception of consumers’ on the city of Glasgow”. |
| Discussion and Title | Your Discussion section is dominated by exploratory findings, which seem disconnected from the main aim of your study (see, e.g., your Abstract). In general, my impression is that your study aims to investigate two issues related to destination image: (1) LA vs. SS associations in time and (2) the differentiation of destination personality between destination inhabitants and non-inhabitants. If so,two things should be improved.  First, the above aims should be reflected in the title, which can refer to Glasgow as an example (case) you use. | This is an excellent point. Section 5.2 has been expanded and renamed “5.2 From Marketers to People: Brand Personality (H2)”. Similarly, Section 5.3 has also been renamed “5.3 Exploratory Analyses”. Although this section has not been shortened, all subsections have been collapsed into paragraphs. The discussion section now comprises three sections 5.1 for H1, 5.2 for H2 and 5.3 for the exploratory hypotheses.  The title has been changed to: “The Language of Marketing Hyperbole and Consumer Perception – the Case of Glasgow”. |
| Discussion | Second, the readers would like to see the research gap: how your findings advance the literature. Were those two issues studied before in the destination context? You do not explain that. For example, in the theoretical background section, you only say, “The present study employs LASS as a theoretical lens to both categorise and interpret the properties generated and provide an additional opportunity to test predictions made by the theory.” | Section 2.3, added: “The present study employs LASS as a theoretical lens to both categorise and interpret the properties generated and provide an additional opportunity to test predictions made by the theory. **To date, no study of destination marketing could be found exploring the role of generation order of terms in word associations studies, nor differences in personal associations versus culturally embedded interpretations of the city concept**.” |
| Conclusions | Conclusions section, you say that “the paper proposed analysing word order in terms of semantic distance, as a means of identifying the activation of different systems in word association tasks.”  Does it mean that some previous studies on tourist destinations already evidenced the LA vs. SS associations in time, and your contribution is only the new method? | No, this aspect had not previously been explored. We have now made that research gap clearer, and followed on from Section 2.3, in Section 7 with:  “To date, the present study appears to be unique in considering the role of dual code theory on word associations applied to destination marketing. One practical implication of this finding (to be tested further in future studies), is that when looking at word association studies, the generation order of terms may indicate two levels of association: an immediate, more generic, and stable image of a city (through common language associations) and a spectrum of situated associations, offering opportunities for a more personalised approach to destination marketing. In short, the present study represents an initial foray into exploring the potential implications of dual code theory on the generation of word associations in the context of destination branding, and that differences may exist in personal associations versus culturally embedded interpretations of the city concept.” |
